# Gait Analysis During Paralysis Recovery in a Rat Incomplete Spinal Cord Injury Model

**DOI:** 10.1002/jsp2.70102

**Published:** 2025-08-06

**Authors:** Misa Toyota, Shion Masuda, Daiki Nohara, Satoru Oba, Mayu Tomomitsu, Momoko Nagai‐Tanima, Tomoki Aoyama

**Affiliations:** ^1^ Kyoto University Human Health Sciences, Graduate School of Medicine Kyoto City Kyoto Prefecture Japan

**Keywords:** contact phase, foot‐off phase, gait analysis, incomplete spinal cord injury, muscle tone

## Abstract

**Background:**

In this study, we aimed to create a rat model of incomplete spinal cord injury and to determine the relationship between muscle tone and gait characteristics during recovery from paralysis. This necessity stems from the need for animal models with motor function dynamics in rehabilitation development for spinal cord injury.

**Methods:**

Thirty‐eight‐week‐old male Sprague‐Dawley rats were divided randomly into two groups: Sham and spinal cord injury groups. Three‐dimensional gait analysis, Hoffman reflex, Basso‐Beatie‐Bresnahan score, muscle wet weight, and histological assessment were performed on postoperative days 3, 7, and 14, respectively.

**Results:**

The incomplete spinal cord injury model showed paralysis recovery over time at postoperative day 14. At ground contact, the ankle plantar flexion angle was higher in the spinal cord injury group than in the Sham group on postoperative day 3; however, it reduced on postoperative day 14. Nevertheless, the ankle plantar flexion angle on the foot‐off phase was significantly higher on postoperative days 3 and 14. The ankle abduction angle in the spinal cord injury group significantly increased over time and was higher than in the Sham group at all time points. Hoffmann reflex results showed that muscle tone was significantly higher in the spinal cord injury group on postoperative day 3 and increased over time. The model's gait was significantly affected by muscle tone changes.

**Conclusion:**

The model provides a valuable tool for studying spastic gait and gait changes associated with improvement in paralysis.

## Graphical abstract

Gait analysis in spinal injury.

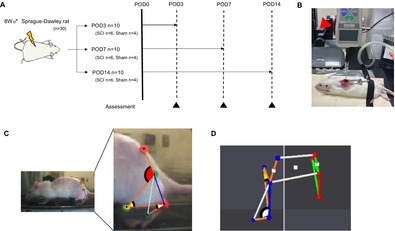

## 1 Introduction

Spinal cord injury (SCI) affects millions of people worldwide with lifelong consequences [1]. SCI is caused primarily by trauma due to motor vehicle accidents or falls [1]. In addition to the direct loss of motor, sensory, and autonomic nervous system function, SCI can cause secondary disabilities at the injury site, significantly reducing the individual's quality of life [2, 3]. In the rehabilitation of SCI, identifying the affected levels and assessing the residual function and functional improvement over time is important [4]. Furthermore, strategies to prevent the disuse of the paralyzed area and promote recovery from paralysis or compensatory strategies using the remaining functions are developed to help patients perform their activities of daily living (ADL) [5]. Furthermore, the management of muscle tone, which occurs in 70% of patients during the recovery process of SCI, is important [6]. The muscle tone can limit ADL, cause fatigue, pain, sleep disturbances, contractures, pressure ulcers, reduce mental health, and reduce QOL [7]. A difficulty in managing muscle tone is the need to balance the beneficial and detrimental effects, as a complete loss of muscle tone can cause muscle weakness [6, 7]. Consequently, an experimental animal model is needed to determine the optimal administration of anti‐spasmodic drugs and the intensity and timing of rehabilitation.

Current animal models of spinal cord injury include complete and incomplete injury models [8]. Spinal cord transection models, which exhibit complete flaccid paralysis, are used in research as rat models of complete SCI [8]. However, the spinal cord transection model differs from the injury mechanism in humans and is unsuitable for assessing the recovery process of paralysis. In contrast, incomplete SCI models, such as contusion (created by dropping dead weights) or compression models (using forceps), which are closer to injury mechanisms in humans, have been used [8, 9]. However, when large weights are dropped, the damage may be extensive, and the degree of paralysis may be uncontrolled. Using these models, gait analysis has been carried out, and many of them are only qualitatively assessed as they cause extensive damage. One of the qualitative evaluations, the Basso–Beattie–Bresnahan (BBB) score, a measure of the degree of motor paralysis to assess motor function recovery, is used in many studies [10]. The BBB score measures hindlimb motor recovery, including joint motion, walking ability, coordination, and trunk stability during free open field walking [11]. It is suitable for assessing the body's ability to move. Muscle tone in models of SCI can be assessed by electrophysiological measurements such as motor evoked potential (MEP), somatosensory evoked potential (SSEP) [12], and Hoffman reflex (H‐reflex) [10, 13]. However, there is a lack of studies on observing the effect of muscle tone on pathological gait during recovery from SCI. These limitations in animal models have hindered the development of drugs and the development of rehabilitation techniques against muscle tone. Therefore, in this study, we aim to develop a rat model of incomplete SCI and to clarify the kinematics of gait and specificity during the recovery process of paralysis.

## 2 Methods

### 2.1 Animals

**FIGURE 1:**
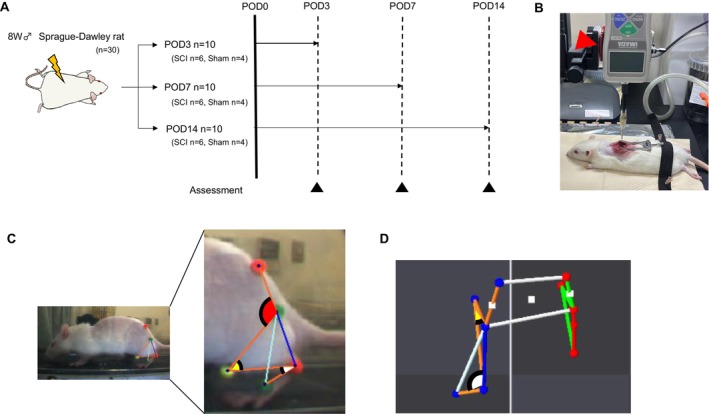
Experimental protocol. (A) Thirty rats were randomly divided into two groups: (A) subject group (Sham, *n* = 4) and a spinal cord injury SCI group (*n* = 6) at POD3, 7, and 14. Black triangle; body weight, H‐reflex, gait analysis, BBB score, and muscle wet weight measurements were performed. (B) The spinal cord was compressed at 1.5 N for 10 s at the 10th thoracic level using a 2 mm wide force gauge (red triangle). (C) Sagittal kinematic analysis using a three‐dimensional motion analysis system. Red arc: hip flexion angle. Yellow arc: knee flexion angle. White arc; plantar flexion angle. (D) Transverse plane kinematic analysis using a three‐dimensional motion analysis system. Yellow arc: knee external rotation angle. White arc: ankle abduction angle. BBB, Basso–Beatie–Bresnahan; POD, post operation day; SCI, spinal cord injury.

Thirty‐eight‐week‐old male Sprague–Dawley (SD) rats (weighing approximately 210–230 g) were classified randomly into two groups: a control (Sham, *n* = 4) and an SCI group (*n* = 6) at postoperative days (POD) 3, 7, and 14 (Figure 1A). The experiment was conducted after deliberation and approval by the Kyoto University Animal Experimentation Committee (Approval Number: 24538) and ARRIVE guidelines.

### 2.2 Surgical Procedure

Surgery was performed under general anesthesia with a triad of anesthetics (1 mL/100 g) administered intraperitoneally. After shaving the back of each rat, the skin was incised, and the subcutaneous fat and paraspinal muscles were separated from the midline to expose the spinous process and vertebral arch of the 10th thoracic vertebra. The most prominent spinous process was used as the index of the 10th thoracic vertebra. The rats in the SCI group were immobilized, and the spinal cord was compressed at 1.5 N for 10 s (Figure 1B) at the 10th thoracic level using a 2 mm wide rod‐mounted digital force gauge (DS2‐44. Imada Co. Ltd., Japan). In the Sham group, the spinal cord was exposed by resecting the spinous process and vertebral arch of the 10th thoracic vertebra; however, no compression was applied. After completing the process, the paraspinal muscles and skin were sutured using a 4‐0 nylon suture (Sp15G04N‐45; Bear Medic, Ibaraki, Japan).

### 2.3 Histological Assessment

Following euthanasia, a 1 cm section of the spinal cord was removed at the site of the crush injury and fixed in 4% paraformaldehyde overnight. Afterward, the spinal cord was paraffin‐embedded. Slides were prepared by slicing at a thickness of 7 μm and stained using hematoxylin and eosin. Assessment of myelin and neuronal Nissl substance was performed by Klüver–Barrera (KB) staining, a double staining of Luxol Fast Blue and Nissl. Similar to previous studies [14], the cavities in the spinal cord larger than 200 μm^2^ observed by KB‐stained images were measured using ImageJ (National Institutes of Health, Bethesda, Maryland, USA) for the area percentage in the full length of the spinal cord.

### 2.4 Body Weight and Muscle Wet Weight Assessment

The body weight (BW) of each rat was measured under general anesthesia at each time point. After euthanasia of the experimental animals, right and left tibialis anterior (TA), extensor digitorum longus (EDL), soleus (SOL), and gastrocnemius (GAS) were harvested, and each muscle's wet weight (MW) was measured. The average values of the right and left muscles were calculated. MW/BW ratio (%) was used for analysis.

### 2.5 H‐Reflex

At POD3, 7, and 14, H and M waves were recorded using an electromyography device (NeuropackS1, Nihon Kohden Co., Tokyo, Japan). Stimulation electrodes were inserted subcutaneously into the endocarp of the rats' hindlimb, and derivation electrodes were placed on the plantar surface of the foot. The potentials evoked by the electrical stimulator were derived from the measurement electrodes, and the maximum values of the H (H_max_) and M waves (M_max_) were recorded. The H_max_/M_max_ ratio (%) was calculated for analysis [13].

### 2.6 BBB Score Scale

The BBB score scale was assessed at POD3, 7, and 14. The BBB score scale is a 22‐point ordinal scale from 0 (no observed hind limb motion) to 21 (normal motion). It is suitable for assessing hind limb motor recovery, including joint motion, walking ability, coordination, and trunk stability during free open‐field walking [11].

### 2.7 Gait Analysis

Kinematic analysis by walking on a treadmill was conducted using a three‐dimensional motion analysis system (Kinema Tracer System, Kissei Comtec, Nagano, Japan) at POD3, 7, and 14. Based on previous studies, the positions were marked with colored hemispherical plastic markers on the superior posterior iliac spine, greater trochanter, knee joint, external phalanx, and fifth metatarsophalangeal joints of the rats [15, 16]. Following filming of the treadmill gait at a comfortable walking speed, the five landmarks were tracked (Figure 1C). Five walking cycles of each foot were used in the analysis. The difference between the maximum and minimum range of motion (ROM) was measured and described as hip flexion–extension (Flex–Ext), knee flexion–extension (Flex–Ext), knee internal–external rotation (Int–Ext Rot), ankle dorsiflexion‐plantar flexion (DF‐PF), and ankle adduction–abduction (Add–Abd) during the entire gait cycle.

To further clarify the gait characteristics, the flexion angles of the knee joint and plantar flexion and abduction of the ankle joint were evaluated at the time of contact and foot‐off phase, which is the time of switching movements. The knee flexion angle was defined as 0° at full flexion, with higher angles indicating greater angular change in the extension direction. The knee external rotation angle was defined as 0° at the mid‐position of external and internal rotation. The ankle plantar flexion angle was measured relative to the ankle length axis with respect to the tibial axis. Furthermore, the fully dorsiflexed position (90° dorsiflexion) was defined as 0° (Figure 1C). The ankle abduction angle was defined as 0° at the mid‐position of abduction [16] (Figure 1D).

### 2.8 Statistical Analysis

Statistical analyses were performed using JMP Pro17 (SAS Institute Inc., Cary, NC, USA). Data normality was confirmed using the Shapiro–Wilk test. Student's *t*‐test was performed to compare the Sham and SCI groups. A two‐way analysis of variance was conducted to examine the effects of time and group factors on the ROM, BBB score, and *H*_max_/*M*_max_ and MW/BW ratio. If significant differences were found, Tukey's HSD test was used as a subtest to confirm changes over time in the SCI group, and comparisons were made between time points. Pearson's correlation coefficient evaluated the correlation between BBB score and range of motion for each joint. *P*‐values were calculated to test the significance of the correlation. Statistical significance was set at 5% for all data.

## 3 Results

### 3.1 Post SCI

**FIGURE 2:**
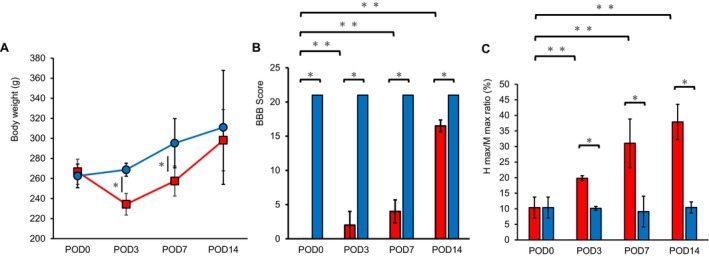
Body weight and change of paralysis. (A) Body weight of SCI (red circle) and Sham rats (blue square). (B) BBB score in SCI rats at POD3, 7, and 14. (C) H_max_/M_max_ in SCI rats at POD3, 7 and 14. *Significant difference between groups, *p* < 0.05. **Significant difference between times *p* < 0.05. Red bar; SCI group. Blue bar; Sham group. Mean ± standard deviation. BBB, Basso–Beatie–Bresnahan; H_max_, maximum values of the H wave; M_max_, maximum values of the M wave; POD, post operation day; SCI, spinal cord injury.

On POD1, mild dysuria and defecation were observed in the SCI group. Abdominal compression was manually applied to promote urination and defecation. No obvious bladder and rectal disturbances were observed after POD2. The body weights of the rats in the SCI group were significantly lower than those in the Sham group at POD3 and POD7; however, by POD14, there was no difference between the two groups (Figure 2A). BBB scores in the SCI group improved over time, leaving only impaired motor coordination during walking at POD14 (Figure 2B). In addition, the results showed significant differences in BBB scores for time factors (*p* < 0.0001), group factors (*p* < 0.0001), and interactions (*p* < 0.0001) in SCI group. *H*_max_/*M*_max_ ratio revealed that muscle tone was significantly higher in the SCI group than in the Sham group on POD3 and increased significantly over time (Figure 2C). There were significant differences in the two‐way analysis of variance on the *H*_max_/*M*_max_ ratio for the time factor (*p* < 0.005), group factor (*p* < 0.0001), and interaction effects (*p* < 0.005).

### 3.2 Muscle Weight

**FIGURE 3:**
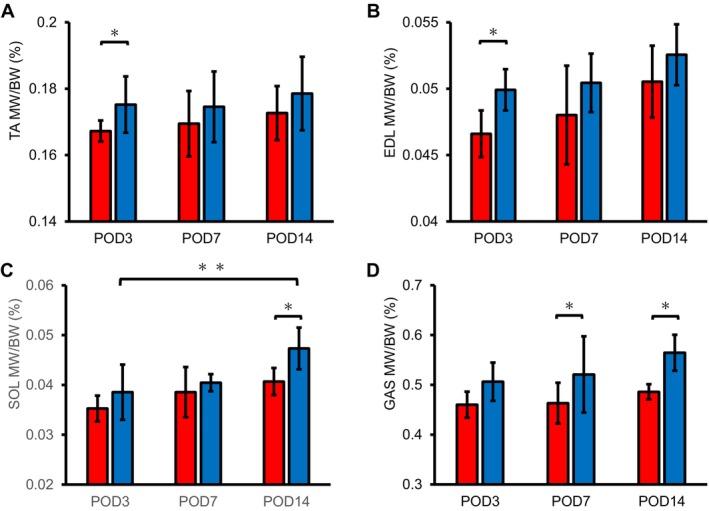
MW/BW ratio in rats with SCI and Sham at POD3, 7, and 14. (A) TA, (B) EDL, (C) SOL, (D) GAS. MW/BW ratio data in the SCI and Sham groups are shown as mean ± standard deviation. *Significant difference between groups *p* < 0.05. **Significant difference between times *p* < 0.05. BW, body weight; EDL, extensor digitorum longus; GAS, gastrocnemius; MW, muscle's wet weight; SCI, spinal cord injury; SOL, soleus; TA, tibialis anterior.

It was lower in the SCI group and could increase over time in both groups (Figure 3). The TA and EDL ratios were notably lower in the SCI group than in the Sham group on POD3 (Figure 3A,B). In addition, SOL on POD14 (Figure 3C) and GAS on POD7 and 14 were substantially lower in the SCI group (Figure 3D). There were significant differences in the two‐way analysis of variance in TA for the interaction effect, SOL for the time factor and group factor, and GAS for the group factor (Table 1).

**TABLE 1:** Two‐way analysis of variance results for MW/BW ratio.

	TA	EDL	SOL	GAS
Day	0.1750	0.505	0.0048*	0.3455
Group	0.0137*	0.5852	0.0215*	0.0016*
Day*group	0.5616	0.1825	0.4717	0.2562

*Note:* *p < 0.05.

Abbreviations: BW, body weight; EDL, extensor digitorum longus; GAS, gastrocnemius; MW, muscle's wet weight; SOL, soleus; TA, tibialis anterior.

### 3.3 Histological Findings of Post‐SCI

**FIGURE 4:**
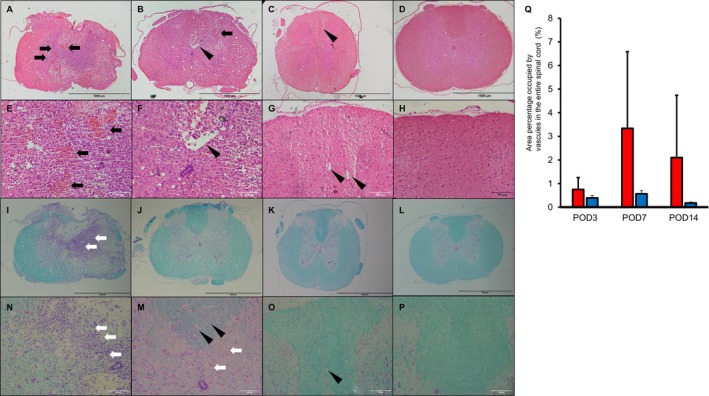
Histological findings of spinal cord tissue. HE staining of the spinal cord site of inflicted damage at POD3 (A, E); POD7 (B, F); POD14 (C, G); and Sham (D, H). KB staining of the spinal cord at POD3 (I, N); POD7 (J, M); POD14 (K, O); and Sham (L, P) are shown. 50× magnification (A–D, I–L), scale bars = 100 μm. 200× magnification (E–H, M–P), scale bars = 100 μm. Black arrows indicate red blood cells. White arrows indicate inflammatory cells. Triangle head indicate cavities. (Q): quantitative assessment of cavities. Red bar; SCI group. Blue bar; Sham group. Mean ± standard deviation. HE, hematoxylin eosin; KB, Klüver–Barrera; POD, post‐operative day; SCI, spinal cord injury.

On POD3, there was a massive disruption of spinal structures extending into the posterior funiculus and gray matter (Figure 4A,I). Hemorrhage (Figure 4E) and numerous inflammatory cells were observed in the gray matter (Figure 4N). On POD7, hemorrhage decreased (Figure 4B,F); however, inflammatory cells remained (Figure 4M). Furthermore, cavity formation was observed in the posterior to lateral funiculus; however, it was reduced in the gray matter (Figure 4F,M). On POD14, inflammatory cells had disappeared (Figure 4C,K) and gray matter structure had recovered (Figure 4K) to an unchanged extent compared to the sham group (Figure 4L). These findings were not observed in the sham group (Figure 4D,H). Cavity formation remained primarily in the posterior funiculus (Figure 4G,O). A comparison of the number of cavities over time showed that they were less common in POD3, with the highest proportion of cavities on POD7, and a slight decrease on POD14 (Figure 4Q).

### 3.4 ROM During Walking

**FIGURE 5:**
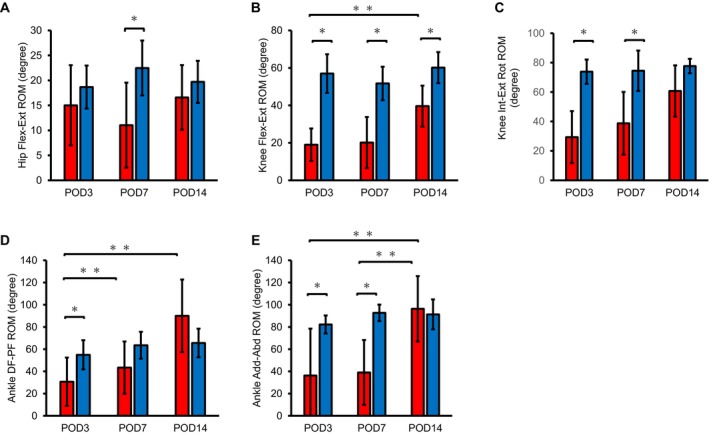
ROM during walking in the SCI and Sham rats at each time point. The ROM of each joint during walking at POD3, 7, and 14 are shown. (A) hip flex‐ext; (B) knee flex‐ext; (C) knee int‐ext rot; (D) ankle DF‐PF and (E) ankle Add–Abd. Red bar: SCI group. Blue bar: Sham group. Mean ± standard deviation. *Significant difference between groups *p* < 0.05. **Significant difference between times, *p* < 0.05. Add–Abd, adduction–abduction; DF‐PF, dorsiflexion‐plantar flexion; Int‐ext rot, internal‐external rotation; Flex‐ext, flexion‐extension; POD, post operation day; ROM, range of motion.

The kinematic analysis showed the ROM of hip Flex–Ext. The SCI group exhibited lower values than in the Sham group only on POD7 (Figure 5A). The knee Flex–Ext ROM at all time points (Figure 5B), and knee Int‐Ext Rot on POD3 and 7 (Figure 5C) were markedly lower in the SCI group than in the Sham group.

In the SCI group, ankle DF‐PF ROM and Add–Abd ROM increased notably from POD7–14 and from POD3–14 (Figure 5D,E). In the SCI group, ankle DF‐PF ROM was significantly lower than in the Sham group at POD7 (Figure 5D), and Add–Abd at POD3 and 7 (Figure 5E). Moderate correlation was noted between ankle DF‐PF, knee joint Int–Ext Rot, and hip Flex‐Ext ROM and BBB scores (*r* = 0.44, 0.42, and 0.41, respectively; Table 2). Significant correlations were noted between BBB scores and the ankle joint Int–Ext Rot and knee joint Flex–Ext ROM (*r* = 0.73, 0.71, respectively; Table 2). These findings indicate an increase in ROM with improvement in paralysis. Furthermore, the two‐way analysis of variance revealed significant differences in ankle DF‐PF ROM regarding longitudinal factors and interactions (Table 3). In addition, significant differences were found in the ankle joint Add–Abd ROM, knee joint Flex–Ext ROM, and knee joint Int–Ext Rot ROM for all longitudinal factors, group factors, and interactions (Table 3). In the SCI group, the ankle DF‐PF and ankle joint Add–Abd ROM significantly increased from POD 7–14 and from POD 3–14 (Figure 5D,E), respectively, as a result of sub testing. In the knee joint, only Flex–Ext ROM significantly expanded from POD 3–14 (Figure 5B).

**TABLE 2:** Correlation between joint ROM and BBB score.

	Ankle		Knee		Hip
	DF‐PF	Add–Abd	Flex–ext	Int–ext rot	Flex–ext
*p*	0.0009	< 0.0001	< 0.0001	0.0179	0.0077
*r*	0.44	0.73	0.71	0.42	0.41

Abbreviations: Add–Abd, adduction–abduction; BBB, Basso–Beatie–Bresnahan; DF‐PF, dorsiflexion‐plantar flexion; Flex–ext, flexion–extension; Int–ext rot, internal–external rotation.

**TABLE 3:** Two‐way analysis of variance results in joint ROM during walking.

	Ankle		Knee		Hip
	DF‐PF	Add–Abd	Flex–ext	Int–ext rot	Flex–ext
Day	0.0011*	0.0127*	0.0018*	0.0024*	0.8263
Group	0.1694	0.0028*	< 0.0001*	0.0279*	0.059
Day*group	0.0051*	0.0156*	0.0494*	0.04695*	0.205

*Note:* *p < 0.05.

Abbreviations: Add–Abd, adduction–abduction; DF‐PF, dorsiflexion‐plantar flexion; Flex–ext, flexion–extension; Int–ext rot, internal–external rotation; ROM, range of motion.

### 3.5 Joint Angles at Contact Phase

**FIGURE 6:**
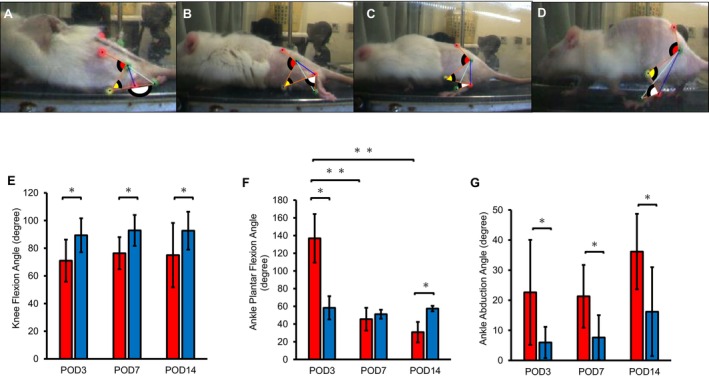
Joint angles at the contact phase. The joint angle was measured at ground contact phase by three‐dimensional motion analysis system. The ankle plantar flexion angle, ankle abduction angle, and knee flexion angle were measured at (A) POD3; (B) POD7; (C) POD14; and (D) Sham; (E) knee flexion angle; (F) ankle plantar flexion angle; (G) ankle abduction angle. POD, post‐operation day; SCI, spinal cord injury. Data at each time point for the SCI and Sham groups in the contact phase are shown as mean ± standard deviation (E–G). Red bar: SCI group. Blue bar: Sham group. *Significant difference between groups *p* < 0.05. **Significant difference between times *p* < 0.05.

At ground contact, the flexion angle of the knee joint was lower at all time points (Figure 6A–C) compared to the Sham group (D,E).

The ankle plantar flexion angle was higher in the SCI group than in the Sham group on POD3 (Figure 6A,D,F); however, it reduced on POD14 (Figure 6C,F). The ankle abduction angle was notably greater in the SCI group at all time points (Figure 6G). The result of the two‐way analysis of variance on ankle plantar flexion angle, ankle abduction, and knee flexion for the time factor (*p* < 0.0001, < 0.0001, and = 0.0105, respectively), group factor (*p* = 0.1407, < 0.0001, and < 0.0001, respectively), and interaction effects (*p* < 0.0001, < 0.0001, and = 0.0742, respectively).

### 3.6 Joint Angles at Foot‐Off Phase

**FIGURE 7:**
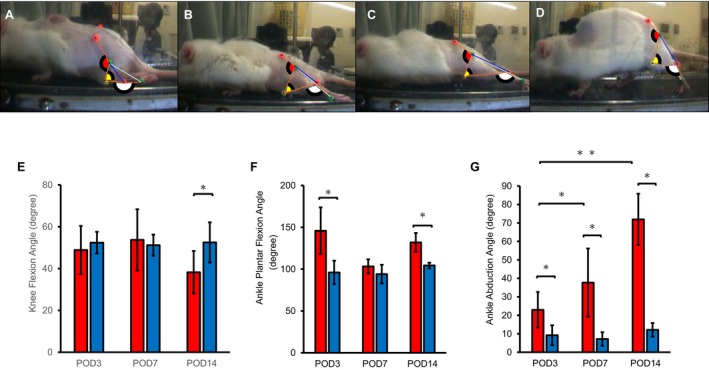
Joint angles at foot off phase. Joint angle was measured at foot‐off phase by a three‐dimensional motion analysis system. The ankle plantar flexion angle, ankle abduction angle, and knee flexion angle were measured at (A) POD3; (B) POD7; (C) POD14; and (D) Sham; (E) knee flexion angle; (F) ankle plantar flexion angle; (G) ankle abduction angle. Data at each time point for the SCI and Sham groups in foot‐off phase are shown as mean ± standard deviation (E–G). Red bar; SCI group. Blue bar; Sham group. *Significant difference between groups *p* < 0.05. **Significant difference between times *p* < 0.05. POD, post operation day; SCI, spinal cord injury.

The knee flexion angle between SCI and Sham groups at foot‐off on POD3 (Figure 7A,D,E) and POD7 was not significantly different (Figure 7B,D,E); however, on POD14, that of the SCI group was significantly lower than the Sham group (Figure 7C–E). The ankle plantar flexion angle was significantly higher in the SCI group than in the Sham group on POD3 (Figure 7A,F) and POD14 (Figure 7B,F). The ankle abduction angle in the SCI group increased significantly over time (Figure 7G) and was higher in the Sham group at all time points (Figure 7G). The result of the two‐way analysis of variance in the ankle plantar flexion angle, ankle abduction, and knee flexion for time factor (*p* < 0.005, < 0.0001, and = 0.3386, respectively), group factor (*p* < 0.0001, < 0.0001, and = 0.0846, respectively), and interaction effects (*p* < 0.005, < 0.0001, and < 0.001, respectively).

## 4 Discussion

Mild weight loss (Figure 2A) and transient dysuria were observed in the SCI model created for this study. BBB scores (Figure 2B) showed that motor paralysis improved in approximately 2 weeks alongside dysuria. Furthermore, improvement in motor paralysis in this model has been shown by changes in muscle weight (Figure 3). The histological finding shows acute to subacute changes in SCI, including the course of inflammatory findings changing over time to fibrosis and cavity formation (Figure 4). Cavity formation, particularly in the posterior funiculus, and changes in gray matter were consistent with the characteristics of previously reported models of incomplete injury [13]. These findings suggest that the present rat model of SCI can approximately replicate the process of recovery from SCI [17, 18, 19]. In addition, MEP and SSEP are widely recognized electrophysiological tools for monitoring motor and sensory pathway integrity [12]. These methods are the gold standard for assessing the corticospinal and sensory tracts; however, they greatly reflect influences from the central nervous system. In this study, H‐reflex measurements were employed in the pictures to clarify the relationship between motor function and muscle tone in neurological recovery post‐SCI (Figure 2C).

The ROM of the lower extremity joints during the gait cycle correlated strongly with the BBB score (Table 1). The observed increased ROM of the ankle joint indicated that, in addition to improvements in lower limb paralysis, the increased muscle output associated with the increased MW/BW ratio of the ankle dorsiflexors, TA, and EDL may have contributed to this change. Furthermore, the muscles that showed an increase in MW exhibited an increased number of muscle nuclei; the number of muscle nuclei correlated positively with the myofiber cross‐sectional area [20], and there was a strong positive correlation between anatomical muscle cross‐sectional area and muscle strength [21, 22].

Increased muscle tone in the early period after spinal cord injury reflected the hyperactivity of reflexive muscle contractions, which is a hallmark of spastic gait [23]. Furthermore, it enhanced the reorganization of spinal circuits and contributed to efficient muscle activity during walking [24]. In this study, muscle tone increased at POD3 before the improvement in paralysis, and this may have contributed to the improved gait (Figure 2C). Previous research stated that stimulating paralyzed muscles to increase muscle tone improves gait [25]. Increased muscle tone may promote muscle contraction around the ankle joint (Figure 5D,E), which spills over to the knee and hip joints as part of the kinetic chain, resulting in an increased ROM of the knee joint (Figure 5A–C). In this study, significant differences in hip flexion angles between the SCI and Sham groups were observed only at POD7. This transient alteration may reflect a compensatory mechanism during the subacute phase, potentially contributing to foot clearance during swing in response to impaired ankle dorsiflexion. Hip kinematics normalization by POD14 indicated that proximal joint adaptation is dynamically regulated depending on the recovery stage and the severity of distal dysfunction. According to previous studies, multiple muscle groups are involved in the knee joint motion, including the quadriceps and hamstrings and gastrocnemius muscles [26]. Therefore, the knee joint motion requires the coordination of many muscle groups and neural circuits during gait. It has been suggested that knee joint recovery is delayed compared to that of the ankle joint. This may be attributed to the time required for muscle group coordination and reorganization of neural circuits. There was no difference in the ankle plantar flexion (Figures 6F and 7F) suggesting that this period was a transitional phase in the recovery process; however, muscle contraction and gait pattern changes continued. Excessive muscle tone may decrease the coordination of joint movements as it is accompanied by the abnormal simultaneous activation of active and antagonistic muscles [27]. Furthermore, it has been established that upper motor neuron lesions result in increased muscle tone and the emergence of synergistic movements [28]. The joint angles at contact and toe‐off on POD14 (Figures 6F and 7F) indicated that excessive hypertonia impaired joint coordination and induced synergistic movement patterns, potentially leading to a gait strategy characterized by a flexor synergy pattern. In contrast to the sustained increase during toe‐off, the observed decrease in ankle plantarflexion angle during foot contact from POD3–14 suggested a phase‐specific modulation of joint mechanics after SCI. One possible explanation is that, in the early postoperative period, tissue edema or inflammation limited ankle mobility, particularly during stance when weight‐bearing occurs. As the edema subsides, joint flexibility may improve, leading to a reduction in plantarflexion angle during contact. No edema or inflammatory findings were observed in the gross foot assessment (data not shown); however, the possibility of edema or inflammation that cannot be confirmed visually cannot be ruled out. Conversely, the persistently elevated angle at toe‐off may reflect increased muscle tone due to spasticity, which becomes increasingly pronounced during the subacute phase. This interpretation is supported by the enhanced H‐reflex responses observed in the same period, suggesting hypertonia. Abduction of the ankle joint is more effective than the mid‐ankle position in enhancing weight bearing by increasing the basal support plane, decreasing joint torque, and decreasing the muscle load to support the body weight [29]. It is suggested that in the early stages of difficult ankle dorsiflexion, the ankle joint may have been in a constant plantar flexion position, and the increased abduction angle may have expanded the support base, reduced joint torque, and compensated for load support (Figures 6G and 7G).

This study had some limitations. Muscle tone using the H‐reflex was only measured in the plantar muscle. For muscle tone assessment in the lower limb, it is desirable to measure more muscles, such as the quadriceps and hamstrings. Long‐term observation could reveal the long‐term effects of muscle tone on gait and muscle tissue, as well as on the regenerating spinal cord. Furthermore, some assessments, including electromyographic and inflammatory markers, were not conducted in this study. Therefore, discussing the effects of the central nervous system and inflammation is impossible. A more comprehensive understanding could be improved if these measurements were carried out. Despite some limitations of this study, the model can contribute to our understanding of spastic gait and to the development of pharmacological and rehabilitation treatments for spastic gait.

## 5 Conclusions

A rat SCI model was developed to examine the relationship between muscle tone and gait characteristics. Motor paralysis, gait, and muscle tone improved at the same time and correlated with each other. Muscle tone initially contributed to an improvement in gait of the paralyzed ankle joint; however, it resulted in gait abnormalities such as abnormal ankle abduction. The rat SCI model created in this study is useful for observing spastic gait and gait changes associated with improvement in paralysis.

## Author Contributions

Misa Toyota contributed to the research design, the acquisition, analysis, and drafting of the paper. Shion Masuda, Daiki Nohara, Mayu Tomomitsu, and Satoru Oba contributed to data interpretation. Momoko Nagai‐Tanima and Tomoki Aoyama supervised the research design and revised paper. All the authors have approved the submitted and final versions.
